# 122. Streptococcus pneumoniae Nasopharyngeal Colonization in a Colombian Cohort of Patients with Comorbid Conditions

**DOI:** 10.1093/ofid/ofac492.200

**Published:** 2022-12-15

**Authors:** Elsa D D Ibáñez-Prada, Cristian C Serrano-Mayorga, Yuli V Fuentes, Julian Lozada, Ingrid G G Bustos, Lina Mendez, Ana M Crispin, Luis Felipe F Reyes

**Affiliations:** Universidad de La Sabana, Chia, Cundinamarca, Colombia; Universidad de La Sabana, Chia, Cundinamarca, Colombia; Universidad de La Sabana, Chia, Cundinamarca, Colombia; Universidad de La Sabana, Chia, Cundinamarca, Colombia; Universidad de La Sabana, Chia, Cundinamarca, Colombia; Clinica Universidad de La Sabana, Chía, Cundinamarca, Colombia; Clinica Universidad de La Sabana, Chía, Cundinamarca, Colombia; Universidad de La Sabana, Chia, Cundinamarca, Colombia

## Abstract

**Background:**

Community-acquired pneumonia (CAP) remains the first cause of infectious death and morbidity worldwide. Nevertheless, its etiological pathogen is only isolated in almost 30% of cases, and its most representative bacteria is *S. pneumoniae*. Some researchers have proposed that the nasopharyngeal colonization by *S. pneumoniae* could risk developing CAP. However, the prevalence of nasopharyngeal colonization in patients with chronic comorbid conditions in Latin America is unknown, where vaccination rates are low. This study aims to determine the rate of *S. pneumoniae* colonization in a Colombian cohort.

**Figure 1.**
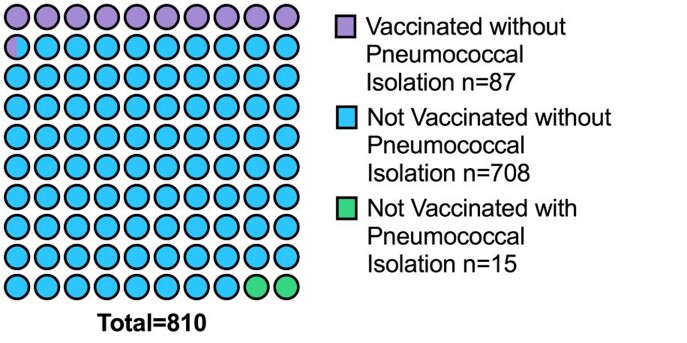
Pneumococcal vaccination and colonization in the whole cohort.

**Methods:**

This is a prospective cohort study in 3 centers in Colombia carried out between 2020 and 2022. According to the world health organization guidelines, the nasopharyngeal aspirate (NPA) samples from each participant were obtained. Samples were incubated with 5% CO2 in blood agar, and MALDI-TOFF identified the colonies obtained. We compared the number of *S. pneumoniae* isolated and stratified by anti-pneumococcal vaccination status.

**Results:**

The cohort was composed of 810 patients. The median [IQR] age was 63 [53-62] and the most common comorbidities were 52.2% [423/810] arterial hypertension, 21.7% [176/810] coronary disease, 19.0% [154/810] congestive heart failure, and 17.4% [141/810] chronic kidney disease. Nasopharyngeal colonization by *S. pneumonie* was only documented in the patients' 2.1% [15/723] (Figure 1). Moreover, 10.7% [87/810] patients from the cohort were vaccinated against *S. pneumoniae*, and none were colonized.

**Conclusion:**

Nasopharyngeal colonization by *S. pneumoniae* in our cohort of adults with chronic comorbidities was low. Notably, all the colonized patients were not vaccinated, at higher risk of developing the invasive pneumococcal disease. More robust vaccination policies should be implemented for adults in Colombia.

**Disclosures:**

**All Authors**: No reported disclosures.

